# Vibration-Based Structural Health Monitoring Using Piezoelectric Transducers and Parametric *t*-SNE

**DOI:** 10.3390/s20061716

**Published:** 2020-03-19

**Authors:** David Agis, Francesc Pozo

**Affiliations:** Control, Modeling, Identification, and Applications (CoDAlab), Department of Mathematics, Escola d’Enginyeria de Barcelona Est (EEBE), Universitat Politècnica de Catalunya (UPC), Campus Diagonal-Besòs (CDB), Eduard Maristany, 16, 08019 Barcelona, Spain; david.agis@upc.edu

**Keywords:** classification, detection, parametric *t*-distributed stochastic neighbor embedding (P-*t*-SNE), piezoelectric transducers (PZTs), principal component analysis (PCA), structural health monitoring (SHM), vibration-based SHM

## Abstract

In this paper, we evaluate the performance of the so-called parametric *t*-distributed stochastic neighbor embedding (P-*t*-SNE), comparing it to the performance of the *t*-SNE, the non-parametric version. The methodology used in this study is introduced for the detection and classification of structural changes in the field of structural health monitoring. This method is based on the combination of principal component analysis (PCA) and P-*t*-SNE, and it is applied to an experimental case study of an aluminum plate with four piezoelectric transducers. The basic steps of the detection and classification process are: (i) the raw data are scaled using mean-centered group scaling and then PCA is applied to reduce its dimensionality; (ii) P-*t*-SNE is applied to represent the scaled and reduced data as 2-dimensional points, defining a cluster for each structural state; and (iii) the current structure to be diagnosed is associated with a cluster employing two strategies: (a) majority voting; and (b) the sum of the inverse distances. The results in the frequency domain manifest the strong performance of P-*t*-SNE, which is comparable to the performance of *t*-SNE but outperforms *t*-SNE in terms of computational cost and runtime. When the method is based on P-*t*-SNE, the overall accuracy fluctuates between 99.5% and 99.75%.

## 1. Introduction

In structural health monitoring (SHM), an important process for engineering structures, many methods have been applied for damage detection. In [1], the use of the Treed Gaussian Process model—a class of powerful switching response surface model—is illustrated in the context of the SHM of bridges. In [2], a SHM methodology, based on the system-identification techniques, is proposed to quantify the structural degradation in laminated composite booms used in satellite application. In [3], it is casted SHM in the context of statistical pattern recognition, and damage or structural changes are detected using two techniques based on time series analysis. In [4], three optimization-algorithm based support vector machines for damage detection in SHM are presented, which are expected to help engineers to process high-dimensional data.

Real-world datasets usually have high dimensionality, and their dimensionality may need to be reduced to facilitate data processing. Dimensionality reduction is the process of reducing the number of high-dimensional variables by obtaining a low-dimensional set of variables. This reduced representation must correspond to the intrinsic information of the data. Dimensionality reduction is very important, because it alleviates undesired properties of high-dimensional spaces, such as “the curse of dimensionality” [5]. In the literature, various dimensionality reduction methods have been proposed: (i) linear methods, such as principal component analysis (PCA) [6,7] and linear discriminant analysis (LDA) [8,9], and (ii) nonlinear methods, such as isometric mapping (ISOMAP) [10,11] and the non-parametric version of *t*-distributed stochastic neighbor embedding (*t*-SNE) [12].

In general, real-world data are likely to be highly nonlinear. Therefore, unsupervised nonlinear dimensionality reduction techniques are widely used in many applications for pattern recognition or classification [13,14], visualization [15,16], and compression [17] of big datasets. Among these types of techniques, *t*-SNE is extensively adopted. However, this method is not designed to support the incorporation of out-of-sample data—that is, the embedding of new data. To avoid this problem, the so-called parametric *t*-SNE (P-*t*-SNE) method [18] was proposed. This method uses a neural network (NN) to learn an explicit parametric mapping function from a high-dimensional data space to a low-dimensional space. Thus, P-*t*-SNE can incorporate out-of-sample data. Another advantage of P-*t*-SNE is that it can be applied to large-scale datasets, while *t*-SNE can only be applied to datasets with a size not greater than the order of thousands.

To address the above-mentioned problems of *t*-SNE, in this work, we propose a strategy that combines PCA and P-*t*-SNE to detect and classify damage to structures to diagnose. This combination is much better than a combination of PCA with *t*-SNE: the proposed method achieves similar embedding performance but with a much lower computational cost and runtime. This was confirmed by our experimental results on an aluminum plate instrumented with four piezoelectric transducers (PZTs). Therefore, the aims of this paper are (i) to compare two approaches (P-*t*-SNE versus *t*-SNE) and (ii) to identify the advantages of the parametric version. To do this, we will use scenarios 1 and 3 from [19], because these two scenarios are two extreme cases. In addition, we will approach the damage detection and classification problem in the frequency domain, as recommended in the same paper [19]. The final classification of the current state of the structure is based on two different voting systems: the so-called majority voting and the sum of the inverse distances [19].

The contributions of this study are summarized in the following list. These contributions mainly refer to the data preprocessing, divided into three parts: data integration, data transformation, and data reduction.

1. Data preprocessing: Data integration. According to [20], there are six different ways to arrange the raw data collected by multiple sensors that collected measurements for several seconds in different experiments. The type of integration of raw data affects the posterior analysis. In this work, we have considered type-*E* unfolding.
2. Data preprocessing: Data normalization. The second step, before data transformation, is the data normalization. We perform the mean-centered group scaling (MCGS), as detailed in [21].
3. Data preprocessing: Data transformation. We build the PCA model (P) so that the normalized data $\overset{˘}{X}$ are transformed into the projected data $T=\overset{˘}{X}P$. Notably, matrices $\overset{˘}{X}$ and T have equal dimensions; hence, no reduction is performed at this stage.
4. Data preprocessing: Data reduction (phase I). We use PCA for the data reduction. The number of principal components ℓ∈N is chosen so that the proportion of the variance explained is greater than or equal to 95%.
5. Data preprocessing: Data reduction (phase II). We now propose P-*t*-SNE, avoiding the limitations of *t*-SNE, as a second phase to reduce the dimensionality from *ℓ* to 2. This is one of the first approaches that use P-*t*-SNE in the field of SHM.
6. Data postprocessing: Classification. For the classification, we propose the majority voting and the sum of the inverse distances, where each actuation phase casts a vote.

As a summary, the contribution of the present work is, precisely, the combination of existing preprocessing methods with a very promising approach: P-*t*-SNE.

The remainder of this paper is structured as follows. In Section 2, we present the P-*t*-SNE method. Section 3 describes the preprocessing of the baseline data, reduction of the global dimension of the data, and creation of the clusters using P-*t*-SNE. Section 4 describes the damage diagnosis procedure. Subsequently, the application of the proposed method is presented in Section 5. Section 6 shows the results. Finally, Section 7 provides our conclusions.

## 2. Parametric *t*-SNE (P-*t*-SNE)

The non-parametric version of *t*-SNE has a huge computational cost of optimization: to map new data, the optimization has to run for the complete set again. To avoid the heavy optimization of the *t*-SNE, the P-*t*-SNE was proposed. P-*t*-SNE is an unsupervised dimensionality reduction technique that learns a parametric mapping between the high-dimensional data space and the low-dimensional latent space, preserving the local structure of the data in the latent space as well as possible.

In the P-*t*-SNE method, the mapping f:X→Y from the high-dimensional space *X* to the low-dimensional space *Y* is parameterized through a feed-forward NN with weights *W*. The NN is trained in such a way that it retains the local structure of the data in the low-dimensional space. There are two main stages in the training procedure:

1. Pretraining with a restricted Boltzmann machine (RBM). RBM is used to construct a pretrained P-*t*-SNE network. The main aim of the pretraining stage is to define an initialization of the model parameters for the next stage.
2. Fine-tuning using the cost function of P-*t*-SNE. In this stage, the weights of the pretrained NN are fine-tuned in such a way that the NN preserves the local structure of the data in the low-dimensional space. In the feed-forward NN, term $q_{ij}$ [12,19] of the *t*-SNE is adapted as follows:
  $$\begin{array}{cc} q_{ij} & =\frac{{\left[1+∥f\left(x_{i}|W\right)-f\left(x_{j}|W\right){∥}^{2}/\alpha \right]}^{-(\alpha +1)/2}}{\sum_{k\neq l}{\left[1+∥f\left(x_{k}|W\right)-f\left(x_{l}|W\right){∥}^{2}/\alpha \right]}^{-(\alpha +1)/2}}, \end{array}$$
  where α denotes the degrees of freedom of the Student’s *t*-distribution.

### Restricted Boltzmann Machine

In this section, a short introduction to RBM [22,23] is given.

**Figure 1:**
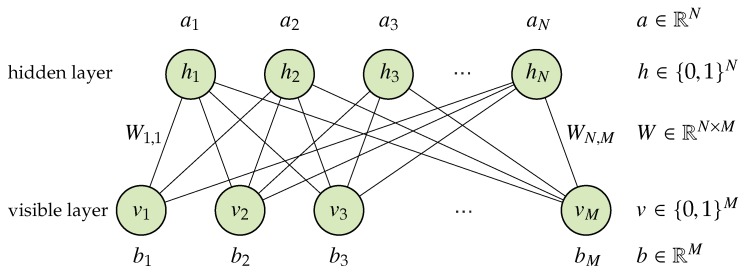
Structure of the RBM.

An RBM is a two-layer stochastic NN. This network consists of a visible, or input, layer (visible nodes v) and a hidden layer (hidden nodes h). Values of the nodes are normally Bernoulli-distributed. Each visible node is connected to all hidden nodes using weighted connections, but there are no intra-layer connections. The structure of the RBM is illustrated in Figure 1.

Boltzmann distribution is specified by the energy function E(v,h), and this distribution gives the joint distribution over all nodes, P(v,h):

$$\begin{array}{cc} E(v,h) & =-\sum_{i,j}W_{ij}v_{i}h_{j}-\sum_{i}b_{i}v_{i}-\sum_{j}a_{j}h_{j},\\ P(v,h) & =\frac{\exp(-E(v,h))}{\sum_{v,h}\exp\left(-E\left(v,h\right)\right)}, \end{array}$$

where $W_{ij}$ is the weight of the connection between a visible node $v_{i}$ and a hidden node $h_{j}$; and $b_{i}$ and $a_{j}$ are the biases of visible and hidden nodes, respectively. Moreover, conditional probabilities $P(v_{i}=1|h)$ and $P(h_{j}=1|v)$ are given by the sigmoid function:

(1) $$\begin{array}{c} P\left(v_{i}=1|h\right)=\frac{1}{1+\exp(-\sum_{j}W_{ij}h_{j}-b_{i})}, \end{array}$$

(2) $$\begin{array}{c} P\left(h_{j}=1|v\right)=\frac{1}{1+\exp(-\sum_{i}W_{ij}v_{i}-a_{j})}. \end{array}$$

The RBM can calculate the values of visible nodes from the values of hidden nodes by Equation (1); similarly, the RBM can calculate the values of hidden nodes from the visible nodes by Equation (2).

The model parameters *W*, *b*, and *a* are learned so that the marginal distribution over the visible nodes under model $P_{\mathrm{model}}\left(v\right)$ is close to the true distribution of data, $P_{\mathrm{data}}\left(v\right)$. In particular, the RBM uses the Kullback–Leibler (KL) divergence to measure the distance between the true distribution $P_{\mathrm{data}}\left(v\right)$ and the distribution based on the model $P_{\mathrm{model}}\left(v\right)$. The gradient of the KL divergence with respect to $W_{ij}$ is given by

$$\begin{array}{c} \frac{\partial KL(P_{\mathrm{data}}||P_{\mathrm{model}})}{\partial W_{ij}}=E{\left[v_{i}h_{j}\right]}_{P_{\mathrm{data}}}-E{\left[v_{i}h_{j}\right]}_{P_{\mathrm{model}}}, \end{array}$$

where $E{\left[v_{i}h_{j}\right]}_{P_{\mathrm{data}}}$ is the expected value under the true distribution, and $E{\left[v_{i}h_{j}\right]}_{P_{\mathrm{model}}}$ is the expected value under the model distribution.

However, the model expectation, $E{\left[v_{i}h_{j}\right]}_{P_{\mathrm{model}}}$, cannot be computed analytically. To avoid computing the model, we follow an approximation: the gradient of a slightly different objective function that is called contrastive divergence (CD) [24]. The CD measures how the model distribution gets away from the true distribution of data through $KL(P_{\mathrm{data}}\left|\right|P_{\mathrm{model}})-KL(P_{1}\left|\right|P_{\mathrm{model}})$, where $P_{1}\left(v\right)$ is the distribution over the visible nodes because the RBM can run for one iteration (that is, one Gibbs sweep) when initialized according to the true distribution. Using standard gradient descent techniques, the CD can be minimized efficiently:

$$\begin{array}{c} E{\left[v_{i}h_{j}\right]}_{P_{\mathrm{data}}}-E{\left[v_{i}h_{j}\right]}_{P_{1}}. \end{array}$$

The second term, $E{\left[v_{i}h_{j}\right]}_{P_{1}}$, is estimated from the samples obtained using Gibbs sampling.

## 3. Baseline Data: Preprocessing and Clustering

In this section, data preprocessing is presented briefly, because a more detailed description can be found in [19]. The preprocessing has three stages: data integration (Section 3.1), data transformation (Section 3.2), and data reduction (Section 3.3). Subsequently, data are organized in clusters in Section 3.4.

### 3.1. Data Integration: Unfolding and Scaling

The collected data contain different response signals measured by sensors on a vibrating structure in the time domain. Under different structural states, multiple observations of these responses are measured. Then, using the fast Fourier transform (FFT) algorithm, these response signals are transformed into the frequency domain. All these observations in the frequency domain are collected in a matrix that is defined as follows:

(3) $$\begin{array}{cc} X=\left(x_{i,j}^{k,l}\right) & =\left[\begin{array}{cccccccccc} x_{1,1}^{1,1} & \cdots & x_{1,1}^{1,L} & x_{1,1}^{2,1} & \cdots & x_{1,1}^{2,L} & \cdots & x_{1,1}^{K,1} & \cdots & x_{1,1}^{K,L}\\ \vdots & \ddots & \vdots & \vdots & \ddots & \vdots & \ddots & \vdots & \ddots & \vdots \\ x_{n_{1},1}^{1,1} & \cdots & x_{n_{1},1}^{1,L} & x_{n_{1},1}^{2,1} & \cdots & x_{n_{1},1}^{2,L} & \cdots & x_{n_{1},1}^{K,1} & \cdots & x_{n_{1},1}^{K,L}\\ x_{1,2}^{1,1} & \cdots & x_{1,2}^{1,L} & x_{1,2}^{2,1} & \cdots & x_{1,2}^{2,L} & \cdots & x_{1,2}^{K,1} & \cdots & x_{1,2}^{K,L}\\ \vdots & \ddots & \vdots & \vdots & \ddots & \vdots & \ddots & \vdots & \ddots & \vdots \\ x_{n_{2},2}^{1,1} & \cdots & x_{n_{2},2}^{1,L} & x_{n_{2},2}^{2,1} & \cdots & x_{n_{2},2}^{2,L} & \cdots & x_{n_{2},2}^{K,1} & \cdots & x_{n_{2},2}^{K,L}\\ \vdots & \ddots & \vdots & \vdots & \ddots & \vdots & \ddots & \vdots & \ddots & \vdots \\ x_{1,J}^{1,1} & \cdots & x_{1,J}^{1,L} & x_{1,J}^{2,1} & \cdots & x_{1,J}^{2,L} & \cdots & x_{1,J}^{K,1} & \cdots & x_{1,J}^{K,L}\\ \vdots & \ddots & \vdots & \vdots & \ddots & \vdots & \ddots & \vdots & \ddots & \vdots \\ x_{n_{J},J}^{1,1} & \cdots & x_{n_{J},J}^{1,L} & x_{n_{J},J}^{2,1} & \cdots & x_{n_{J},J}^{2,L} & \cdots & x_{n_{J},J}^{K,1} & \cdots & x_{n_{J},J}^{K,L} \end{array}\right]\in M_{\left(n_{1}+\cdots +n_{J}\right)\times \left(K\cdot L\right)}\left(R\right), \end{array}$$

where K∈N is the number of sensors and k=1,…,K identifies the sensor that is measuring; L∈N is the number of components in each signal and l=1,…,L indicates the *l*-th measurement in the frequency domain; J∈N is the number of different structural states that are considered and j=1,…,J represents the structural state that is measured; finally, $n_{j},\;j=1,\ldots ,J$, is the number of observations per structural state and $i=1,\ldots ,n_{j}$ is the *i*-th observation related to the *j*-th structural state.

Matrix X in Equation (3) is a particular unfolded version of a 3-dimensional $(n_{1}+\cdots +n_{J})\times K\times L$ data matrix, where the first dimension is observation, the second dimension is sensor, and the third dimension is time. Numerous approaches have been proposed to handle 3-dimensional matrices. The most widely adopted approaches are based on the unfolding of these matrices. There are six alternative ways of arranging a 3-dimensional data matrix [20] that affect the performance of the overall strategy, and we have considered type *E* in this work, because type-*E* unfolding simplifies the study of variability among samples.

Matrix X in Equation (3) is rescaled through MCGS [21] because of the different magnitudes and scales in the measurements.

### 3.2. Data Transformation

Data transformation means applying a particular mathematical function. The transformation that we apply in this study is PCA, because the final aim is dimensionality reduction. We build the PCA model, P, so that the normalized data X using MCGS, $\overset{˘}{X}$, are transformed to the projected data $T=\overset{˘}{X}P$. Notably, matrices $\overset{˘}{X}$ and T have equal dimensions; hence, no reduction is performed at this stage.

### 3.3. Data Reduction: PCA and P-*t*-SNE

In this work, we use two methods of data reduction. On the one hand, we apply PCA to represent the normalized matrix $\overset{˘}{X}$ in a new space with reduced dimensions and without a significant loss of information. On the other hand, we apply P-*t*-SNE as a 2-dimensional representation technique. These two approaches are combined to reduce the data complexity, computational effort, and time.

### 3.4. Clustering Effect

The first dimensionality reduction is performed using PCA. Specifically, for $n=n_{1}+\cdots +n_{J}$ observations, the rows of matrix X in Equation (3) under *J* different structural states are projected and transformed into a lower-dimensional space.

Next, the second dimensionality reduction is applied to the projected and transformed data using P-*t*-SNE. The aim is to find a collection of 2-dimensional points that represent the projected and transformed data by PCA with no explicit loss of information and preserve the local structure of this dataset. After the application of P-*t*-SNE, we expect to observe *J* clusters related to *J* different structural states.

As mentioned at the beginning of Section 3, see [19] for more details on these stages.

## 4. Structure to Diagnose: Damage Detection and Classification Procedure

In this section, we describe the vibration-based damage detection and classification procedure to diagnose a new structure.

For damage detection and classification, a single observation of the current structure is required to diagnose it. The collected data are composed of different response signals measured by *K* sensors and *L* components in each signal, as in Equation (3). When these measures are obtained in the frequency domain, we build a new data vector z:

$$\begin{array}{c} z^{⊤}=\left[\begin{array}{cccccccccc} z^{1,1} & \cdots & z^{1,L} & z^{2,1} & \cdots & z^{2,L} & \cdots & z^{K,1} & \cdots & z^{K,L} \end{array}\right]\in R^{K\cdot L}. \end{array}$$

### 4.1. Scaling (MCGS)

First, we have to scale the row vector $z^{⊤}$ to define a scaled row vector ${\overset{˘}{z}}^{⊤}$:

(4) $$\begin{array}{c} {\overset{˘}{z}}^{k,l}=\frac{z^{k,l}-\mu^{k,l}}{\sigma^{k}},\;k=1,\ldots ,K,\;l=1,\ldots ,L, \end{array}$$

where $\mu^{k,l}$ is the arithmetic mean of all the elements in the [(k−1)L+l]-th column of matrix X in Equation (3) (that is, the *l*-th column of the *k*-th sensor) and $\sigma^{k}$ is the standard deviation of all measurements of the *k*-th sensor relative to the mean value $\mu^{k}$ (the arithmetic mean of all the measurements of the *k*-th sensor).

### 4.2. Projection (PCA)

The scaled row vector ${\overset{˘}{z}}^{⊤}\in R^{K\cdot L}$ is projected into the space spanned by the first *ℓ* principal components in $P_{\ell }$ through the vector-to-matrix multiplication:

$$\begin{array}{c} x^{n+1}={\overset{˘}{z}}^{⊤}\cdot P_{\ell }\in R^{\ell }. \end{array}$$

Notably, the vector containing the data of the structure to be diagnosed initially has dimension K·L but later dimension *ℓ*. We add this new point to the projected and transformed data by PCA ($X=\left\{x^{1},\ldots ,x^{n}\right\}\subset R^{\ell }$) to define a new set:

(5) $$\begin{array}{c} X^{\prime }=\left\{x^{1},\ldots ,x^{n}\right\}\cup \left\{x^{n+1}\right\}=\left\{x^{1},\ldots ,x^{n},x^{n+1}\right\}\subset R^{\ell }, \end{array}$$

where

$$\begin{array}{c} x^{i}=e_{i}^{⊤}\overset{˘}{X}P_{\ell },\;i=1,\ldots ,n \end{array}$$

and $e_{i}\in R^{n}$ is the *i*-th element of the canonical basis. The network is trained with $\left\{x^{1},\ldots ,x^{n}\right\}$. Then, $\left\{x^{n+1}\right\}$ will be passed through the trained network.

### 4.3. P-*t*-SNE and Final Classification

Finally, we apply P-*t*-SNE to the *ℓ*-dimensional set $X^{\prime }$ in Equation (5) to find a collection of 2-dimensional map points $Y^{\prime }=\left\{y^{1},\ldots ,y^{n},y^{n+1}\right\}\subset R^{2}$ that represent the original set $X=\left\{x^{1},\ldots ,x^{n}\right\}\subset R^{\ell }$ (the data projected and transformed by PCA) with no explicit loss of information and retaining the local structure of this set. Furthermore, the map point $y^{n+1}$, associated with the data point $x^{n+1}$, is included. That is, the embedded data are constructed applying the trained network: input $X^{\prime }$ and output $Y^{\prime }$. We expect to observe the same *J* clusters related to the *J* different structural states.

For each cluster, we calculate its centroid: the mean of the values of the data points in the cluster. For instance, the centroid associated with the first structural state is

$$\begin{array}{c} Y_{1}^{\prime }:=\frac{1}{n_{1}}\sum_{i=1}^{n_{1}}y^{i}=\frac{y^{1}+\cdots +y^{n_{1}}}{n_{1}}\in R^{2}. \end{array}$$

**Figure 2:**
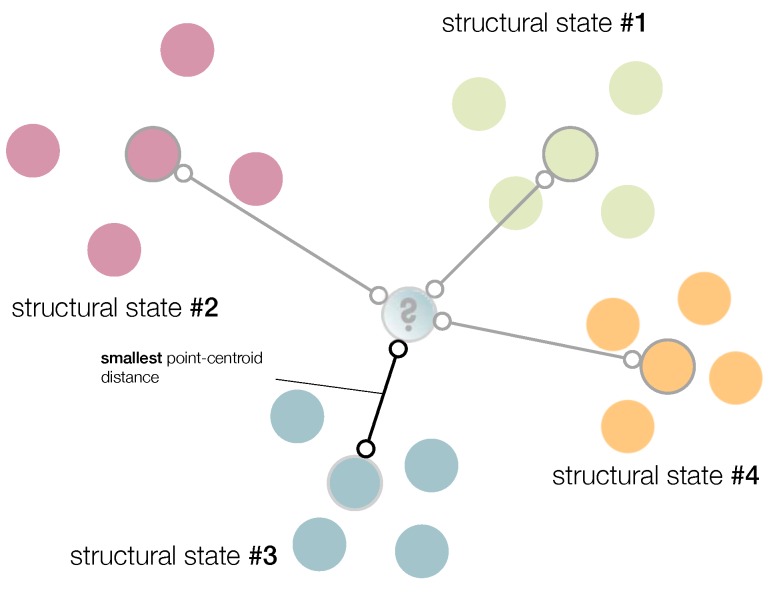
Current structure to diagnose is associated with the structural state with the smallest point-centroid distance.

In general, the centroid associated with the *j*-th structural state, j=1,…,J, is the 2-dimensional point defined as

(6) $$\begin{array}{c} Y_{j}^{\prime }:=\frac{1}{n_{j}}\sum_{i=1}^{n_{j}}y^{\left(\sum_{m=0}^{j-1}n_{m}\right)+j}\in R^{2},\;j=1,\ldots ,J, \end{array}$$

where $n_{0}=0$. As a result, the current structure to diagnose is associated with the *j*-th structural state if

$$\begin{array}{c} j=\arg\min_{j=1,\ldots ,J}{∥Y_{j}^{\prime }-y^{n+1}∥}_{2}, \end{array}$$

that is, if the minimum distance between $y^{n+1}$ and each centroid corresponds to the Euclidean distance between $Y_{j}^{\prime }$ and $y^{n+1}$. We call this approach the smallest point-centroid distance (see Figure 2).

**Figure 3:**
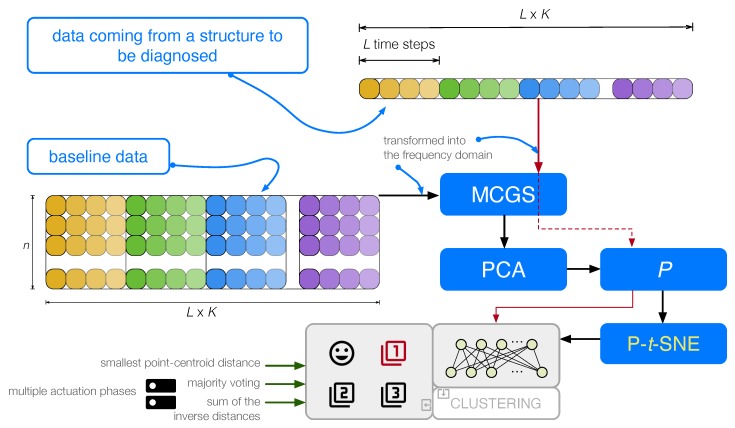
Flowchart of the proposed procedure. Data coming from a structure are first scaled by MCGS and then projected into the PCA model. Finally, P-*t*-SNE is applied to generate the clusters that will be used in the vibration-based detection and classification of structural changes.

The proposed procedure is shown in Figure 3.

## 5. Application of the SHM System on an Aluminum Plate with Four PZTs

In this study, we reuse the structure and experiment from [19]. Therefore, we can use this structure as a benchmark to compare our results.

### 5.1. Structure

**Figure 4:**
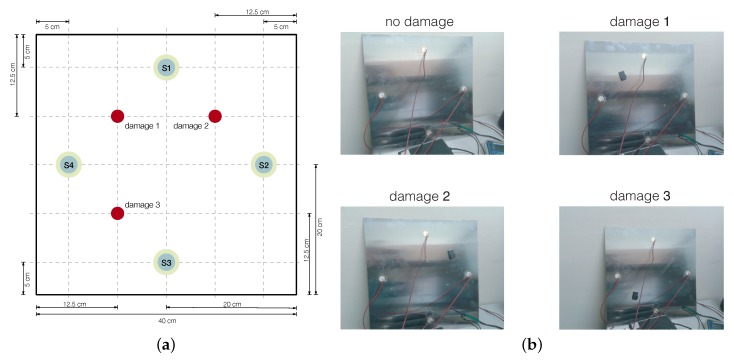
(**a**) Aluminum plate with four piezoelectric sensors (S1, S2, S3, and S4); (**b**) the four structural states considered.

A square aluminum plate was manufactured to demonstrate the accuracy of the vibration-based method of damage detection and classification presented in Section 3 and Section 4. The dimension of the plate is 40×40×0.2 cm. The plate is instrumented with four PZTs and a mass of 17.2916 g is introduced to simulate the damage in a non-destructive way, producing changes in the propagated wave (see Figure 4a). Each PZT can work in two modes: excite the aluminum plate (actuator mode) with a burst signal or detect a time-varying mechanical response (sensor mode). The location of the mass defines each damage, and J=4 structural states are considered: healthy state and three different types of damage (Figure 4b). The plate is isolated from the vibration and noise in the laboratory (Figure 4b).

### 5.2. Scenarios and Actuation Phases

Unlike [19], in this study, the experimental setup includes only two scenarios (the two extreme cases) to determine the performance of the proposed method:

- **Scenario 1**. The signals are obtained using a short wire (0.5 m) from the digitizer to the piezoelectric sensors. Next, the Savitzky–Golay [25] algorithm is used to filter these signals after adding white Gaussian noise. This filter is used to smoothen the data.
- **Scenario 2**. The signals are obtained using a long wire (2.5 m) from the digitizers to the piezoelectric sensors. Signals are also filtered with the Savitzky–Golay algorithm.

As discussed in Section 5.1, there are four PZTs (S1, S2, S3, and S4) that excite the plate and collect the measured signal. This sensor network works in what we call actuation phases. In each actuation phase, a PZT is used as an actuator (which excites the plate with the burst signal), and the rest of the PZTs are used as sensors (which measure signals). Therefore, we have as many actuation phases as sensors: in actuation phase 1, S1 is used as the actuator and the rest of PZTs are used as sensors; in actuation phase 2, S2 is used as the actuator and the rest of PZTs are used as sensors; and so on.

### 5.3. Data Collection

Given a certain scenario, as the two defined in Section 5.2, four matrices X[φ],φ=1,…,4, are obtained, one for each actuation phase. Each matrix X[φ],φ=1,…,4, is constructed as follows:

- $n_{1}=n_{2}=n_{3}=n_{4}=25$ observations are performed for each of the four structural states. Therefore, each matrix X[φ],φ=1,…,4, contains 100 rows: $n_{1}+n_{2}+n_{3}+n_{4}=25\cdot 4$. Specifically, the first 25 rows represent the healthy state, the next 25 are observations with damage 1, and so on.
- For each actuation phase φ,φ=1,…,4, K=3 PZTs working as sensors are measured during 60000 time instants. Next, these measurements are transformed into the frequency domain. As a result, the number of columns of matrix X[φ],φ=1,…,4, is equal to K·L=3·((60000/2)+1)=90003.

Therefore, the matrix that collects all observations under the different structural states in the frequency domain is as follows (see Equation (3); here, L=30001 and J=4):

(7) $$\begin{array}{cc} \; & X\left[\varphi \right]=\left(x{\left[\varphi \right]}_{i,j}^{k,l}\right)\in M_{100\times 90003}\left(R\right). \end{array}$$

The damage detection and classification method described in Section 3 and Section 4 can be applied to each matrix X[φ],φ=1,…,4, in Equation (7), leading to one classification per actuation phase. Then, we will use these four classifications to obtain a final decision based on the four actuation phases. This strategy will be detailed in Section 5.5.

### 5.4. κ-Fold Nonexhaustive Leave-*p*-Out Cross-Validation

The proposed approach is evaluated by comparing test data (the new observations in unknown state under the same conditions) with baseline data (data from the structure under the four different structural states). For that purpose, the κ-fold nonexhaustive leave-*p*-out cross-validation [19] is used. Hence, the sum of all elements in the confusion matrices that are presented in Section 6 is equal to 400. We use the notation **𝔛** to represent the matrix that is used as the baseline to build the model. Matrix **𝔛** has 20 rows: five for each structural state.

### 5.5. Damage Detection and Classification

The strategy for damage detection and classification is as follows: the classification will be based on the four matrices X[1],X[2],X[3], and X[4], defined in Equation (7), with κ-fold nonexhaustive leave-*p*-out cross-validation. Each actuation phase will cast a vote that will determine the final classification.

In this strategy, the next steps are followed:

- **Step 1**. The data matrix **𝔛** is scaled by MCGS, obtaining matrix $\overset{˘}{𝔛}$.
- **Step 2**. PCA is applied to matrix $\overset{˘}{𝔛}$, obtaining the PCA model P.
- **Step 3**. The reduced PCA model $P_{\ell }$ is chosen such that the proportion of variance explained is at least 95%.
- **Step 4**. An observation $z^{⊤}\in R^{3\cdot 30001}=R^{90003}$ of the current structure-to-diagnose is needed. Then, $z^{⊤}$ is scaled by Equation (4) to obtain ${\overset{˘}{z}}^{⊤}$, which is projected into the space spanned by the first *ℓ* principal components in $P_{\ell }$.
- **Step 5**. Dataset $X^{\prime }=\left\{x^{1},\ldots ,x^{20}\right\}\cup \left\{x^{21}\right\}=\left\{x^{1},\ldots ,x^{20},x^{21}\right\}\subset R^{\ell }$ is defined by Equation (5). The network is trained with $\left\{x^{1},\ldots ,x^{20}\right\}$. Then, $\left\{x^{21}\right\}$ will be passed through the trained network.
- **Step 6**. P-*t*-SNE is applied to $X^{\prime }$ to find a set of 2-dimensional points: $Y^{\prime }=\left\{y^{1},\ldots ,y^{20},y^{21}\right\}\subset R^{2}$. Thus, the embedded data are constructed using the trained network: input $X^{\prime }$ and output $Y^{\prime }$.
- **Step 7**. J=4 clusters are obtained, one per structural state. These clusters are formed by the 2-dimensional points: $\left\{y^{1},\ldots ,y^{5}\right\}\subset Y^{\prime }$, related to the healthy state; $\left\{y^{6},\ldots ,y^{10}\right\}\subset Y^{\prime }$, related to damage 1; $\left\{y^{11},\ldots ,y^{15}\right\}\subset Y^{\prime }$, related to damage 2; and $\left\{y^{16},\ldots ,y^{20}\right\}\subset Y^{\prime }$, related to damage 3. Centroid $Y_{j}^{\prime },\;j=1,\ldots ,J$, associated with the *j*-th structural state is calculated by Equation (6).
- **Step 8**. With the information given by the four actuation phases, the structure that must be diagnosed is finally classified considering two approaches: majority voting and sum of the inverse distances. For details of both approaches, see [19].

## 6. Results

In this section, confusion matrices summarize the results of the damage detection and classification method presented in Section 3 and Section 4 and detailed in Section 5.3, Section 5.4 and Section 5.5. The results for each scenario are shown in different subsections. Four different structural states are considered in both scenarios:

- The healthy state (D0)—the aluminum plate with no damage;
- Three states with damage (D1, D2, and D3)—the aluminum plate with an added mass at the positions indicated in Figure 4.

Five iterations (κ=5) of a nonexhaustive leave-*p*-out cross-validation, with p=80, are performed to validate the damage detection and classification method. At each iteration, 80 observations are considered: 20 observations per structural state (D0,D1,D2, and D3). Therefore, the sum of all elements in the confusion matrices is equal to 5·80=400.

Two different confusion matrices are shown for each of the two scenarios:

- **Majority voting**. The damage detection and classification method is applied to the four matrices X[φ],φ=1,…,4. Each actuation phase emits a vote, and the final classification is made by the majority voting [19].
- **Sum of the inverse distances**. The damage detection and classification method is applied to the four matrices X[φ],φ=1,…,4. Each actuation phase emits a vote, and the final classification is made using the maximum sum of the inverse distances [19].

Finally, we have included the confusion matrices for *t*-SNE, to compare its performance with P-*t*-SNE.

### 6.1. Scenario 1

In this section, we describe the results for **Scenario 1** from Section 5.2. Table 1 and Table 2 show the four confusion matrices. The green background cells correspond to observations that are correctly classified, while the red background cells represent the misclassifications. The color intensity (green or red) is related to the proportion of correct or wrong decisions.

With P-*t*-SNE and the majority voting (Table 1), the overall accuracy is very good. Specifically, 398 out of 400 observations have been correctly classified, which corresponds to the overall accuracy of 99.5%. With *t*-SNE, the overall accuracy is 100% (Table 1).

Using the sum of the inverse distances (Table 2) for the P-*t*-SNE-based damage detection and classification, 399 out of 400 observations have been correctly classified; hence, the overall accuracy is 99.75%. For *t*-SNE, 400 out of 400 observations have been correctly classified (100% accuracy).

Furthermore, other metrics are calculated. The most common metrics for choosing the best solution in a binary classification problem are as follows:

- *Accuracy*, defined as the proportion of true results among the total number of cases examined.
- *Precision* or *positive predictive value* (PPV) that attempts to answer the question “what proportion of positive identifications is actually correct?”.
- *Sensitivity* or *true positive rate* (TPR) that measures the proportion of actual positives that are correctly identified as such.
- *$F_{1}$ score*, defined as the harmonic mean of PPV and TPR.
- *Specificity* or *true negative rate* (TNR) that measures the proportion of actual negatives that are correctly identified as such.

These metrics are easy to calculate for binary and multiclass classification problems [26]. When the classification problem is multiclass, as in the current work, according to [27,28], the result is the average obtained by adding the result of each class and dividing over the total number of classes.

In all cases (P-*t*-SNE, *t*-SNE, the majority voting, and the sum of the inverse distances), these five metrics vary between 99.5% and 100% (Table 3 and Table 4).

As can be seen, both parametric and non-parametric approaches obtain practically the same results. However, P-*t*-SNE dramatically reduces the processing time: it decreases from 40 min and 15 s (*t*-SNE) to 2 min and 34 s (P-*t*-SNE) on an Intel Core i7 4.20 GHz computer with 32 GB RAM. Using P-*t*-SNE, a decision is made in just a few milliseconds. The reduced time (2 min and 34 s) includes both the pretraining and the fine-tuning of the NN, as well as the classification of the current state of the structure. The total computational cost of P-*t*-SNE is reduced by approximately 94% compared with *t*-SNE.

### 6.2. Scenario 2

In this section, we describe the results for Scenario 2 from Section 5.2. Table 5 and Table 6 show the four confusion matrices.

With P-*t*-SNE and the majority voting (Table 5), the overall accuracy is also very good. Specifically, 398 out of 400 observations have been correctly classified; this corresponds to the overall accuracy of 99.5%. With *t*-SNE and the majority voting, the overall accuracy is 100% (Table 5).

Using the maximum sum of the inverse distances to take a final decision (Table 6) with P-*t*-SNE, 399 out of 400 observations have been correctly classified (the overall accuracy of 99.75%). With *t*-SNE, 400 out of 400 observations have been correctly classified (the overall accuracy of 100%).

In the non-parametric approach, using the majority voting and the sum of the inverse distances, the five metrics presented in Section 6.1 achieve 100%. In contrast, in the parametric approach, these metrics slightly decrease (between 0.1% and 0.5%, see Table 7 and Table 8).

Again, as in scenario 1, parametric and non-parametric methods obtain similar results. However, as before, the P-*t*-SNE approach dramatically reduces the processing time: from 42 min and 1 s (*t*-SNE) to 2 min and 32 s (P-*t*-SNE). As in the previous scenario, the total computational cost of P-*t*-SNE is reduced by approximately 94% compared with *t*-SNE.

### 6.3. Repeatability

**Figure 5:**
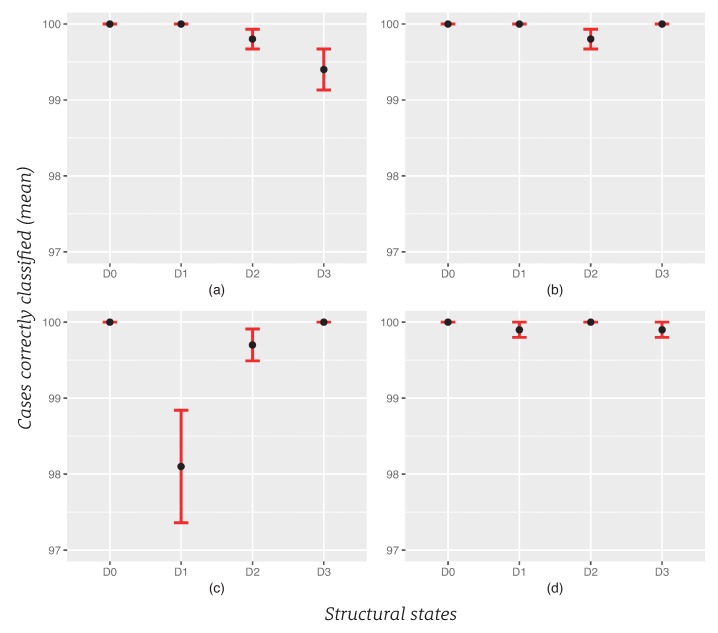
Repeatability of the SHM strategy (10 times), graphs with error bars: (**a**) scenario 1 and majority voting approach; (**b**) scenario 1 and sum of the inverse distances approach; (**c**) scenario 2 and majority voting approach; (**d**) scenario 2 and sum of the inverse distances approach. D0: healthy state of the structure; D1,D2, and D3: added masses at the positions indicated in Figure 4.

We use error bars to give the reader a general idea of the uncertainty in the results. Figure 5 shows the mean of each structural state with error bars representing the standard error. As it can be seen both in Figure 5 and in Table 9, Table 10, Table 11 and Table 12, the standard error is very small, when we repeat the procedure 10 times.

## 7. Conclusions

In this paper, we proposed an SHM strategy for the detection and classification of structural changes combining PCA and P-*t*-SNE. We evaluated the proposed method with experimental data. The obtained results show that its performance is very good, given its high classification accuracy. In addition, we have compared the parametric version of *t*-SNE with the non-parametric version.

According to the results shown in Section 6.1 and Section 6.2, the performance is very satisfactory and similar in both approaches: P-*t*-SNE and *t*-SNE. However, in terms of processing time, it is better to make a decision considering the P-*t*-SNE-based damage detection and classification rather than working with the *t*-SNE-based method: although the non-parametric approach slightly outperforms the parametric approach, the parametric approach can reduce the total computational cost by approximately 94%. Hence, P-*t*-SNE can classify a current structure in just a few milliseconds. This is the first indication that P-*t*-SNE is better compared with the *t*-SNE. Other advantages of using P-*t*-SNE are as follows:

1. P-*t*-SNE can handle large-scale datasets, while *t*-SNE can only handle datasets with a size not greater than the order of thousands.
2. The *t*-SNE method requires an extremely large computational cost for the optimization: to map a new data sample, the optimization has to run for the whole dataset again. However, P-*t*-SNE can learn from the training data and be applied when a new observation arises; hence, it can work with real-time observations. Therefore, the parametric approach can make inferences about new samples to be diagnosed without having to recalculate everything; the model predicts on out-of-sample data.

Based on the foregoing, and seeing the strong performance of the P-*t*-SNE-based approach, we conclude that it is better to work with the parametric version of *t*-SNE than with the non-parametric version.

Many possible fields of application exist. For example, in aeronautics, parts of an airplane (wings or fuselage) can be simulated with similar aluminum plates; for wind turbines, this methodology can be applied to detect damage and faults. In general, if a sensor network can be installed in a structure, and various actuation phases can be defined, the proposed approach can be considered.

In our future work, we contemplate to apply the proposed methodology to handle imbalanced data, as well as to determine its effectiveness in different environmental and operational conditions. 

## Figures and Tables

**Table 1:** Confusion matrix of the application of P-*t*-SNE and *t*-SNE damage detection and classification method presented in Section 3 and Section 4: scenario 1 and the majority voting approach.

	Predicted	P-*t*-SNE				*t*-SNE			
True		*D*0	*D*1	*D*2	*D*3	*D*0	*D*1	*D*2	*D*3
*D*0		100	0	0	0	100	0	0	0
*D*1		0	100	0	0	0	100	0	0
*D*2		0	0	100	0	0	0	100	0
*D*3		0	0	2	98	0	0	0	100

^1^*D*0: healthy state of the structure; *D*1, *D*2, and *D*3: added masses at the positions indicated in Figure 4.

**Table 2:** Confusion matrix of the application of P-*t*-SNE and *t*-SNE damage detection and classification method presented in Section 3 and Section 4: scenario 1 and the sum of the inverse distances approach.

	Predicted	P-*t*-SNE				*t*-SNE			
True		*D*0	*D*1	*D*2	*D*3	*D*0	*D*1	*D*2	*D*3
*D*0		100	0	0	0	100	0	0	0
*D*1		0	100	0	0	0	100	0	0
*D*2		0	0	99	1	0	0	100	0
*D*3		0	0	0	100	0	0	0	100

^1^*D*0: healthy state of the structure; *D*1, *D*2, and *D*3: added masses at the positions indicated in Figure 4.

**Table 3:** Accuracy, PPV, TPR, *F*_1_ score, and TNR of the application of P-*t*-SNE and *t*-SNE damage detection and classification method presented in Section 3 and the Section 4: scenario 1 and the majority voting approach.

	P-*t*-SNE	*t*-SNE
Accuracy	99.8%	100.0%
PPV	99.5%	100.0%
TPR	99.5%	100.0%
*F*_1_ score	99.5%	100.0%
TNR	99.8%	100.0%

**Table 4:** Accuracy, PPV, TPR, *F*_1_ score, and TNR of the application of P-*t*-SNE and *t*-SNE damage detection and classification method presented in Section 3 and Section 4: scenario 1 and the sum of the inverse distances approach.

	P-*t*-SNE	*t*-SNE
Accuracy	99.9%	100.0%
PPV	99.8%	100.0%
TPR	99.8%	100.0%
*F*_1_ score	99.7%	100.0%
TNR	99.9%	100.0%

**Table 5:** Confusion matrix of the application of P-*t*-SNE and *t*-SNE damage detection and classification method presented in Section 3 and Section 4: scenario 2 and the majority voting approach.

	Predicted	P-*t*-SNE				*t*-SNE			
True		*D*0	*D*1	*D*2	*D*3	*D*0	*D*1	*D*2	*D*3
*D*0		100	0	0	0	100	0	0	0
*D*1		1	99	0	0	0	100	0	0
*D*2		1	0	99	0	0	0	100	0
*D*3		0	0	0	100	0	0	0	100

^1^*D*0: healthy state of the structure; *D*1, *D*2, and *D*3: added masses at the positions indicated in Figure 4.

**Table 6:** Confusion matrix of the application of P-*t*-SNE and *t*-SNE damage detection and classification method presented in Section 3 and Section 4: scenario 2 and the sum of the inverse distances approach.

	Predicted	P-*t*-SNE				*t*-SNE			
True		*D*0	*D*1	*D*2	*D*3	*D*0	*D*1	*D*2	*D*3
*D*0		100	0	0	0	100	0	0	0
*D*1		1	99	0	0	0	100	0	0
*D*2		0	0	100	0	0	0	100	0
*D*3		0	0	0	100	0	0	0	100

^1^*D*0: healthy state of the structure; *D*1, *D*2, and *D*3: added masses at the positions indicated in Figure 4.

**Table 7:** Accuracy, PPV, TPR, *F*_1_ score, and TNR of the application of P-*t*-SNE and *t*-SNE damage detection and classification method presented in Section 3 and Section 4: scenario 2 and the majority voting approach.

	P-*t*-SNE	*t*-SNE
Accuracy	99.8%	100.0%
PPV	99.5%	100.0%
TPR	99.5%	100.0%
*F*_1_ score	99.5%	100.0%
TNR	99.8%	100.0%

**Table 8:** Accuracy, PPV, TPR, *F*_1_ score, and TNR of the application of P-*t*-SNE and *t*-SNE damage detection and classification method presented in Section 3 and Section 4: scenario 2 and the sum of the inverse distances approach.

	P-*t*-SNE	*t*-SNE
Accuracy	99.9%	100.0%
PPV	99.8%	100.0%
TPR	99.8%	100.0%
*F*_1_ score	99.7%	100.0%
TNR	99.9%	100.0%

**Table 9:** Repeatability of the SHM strategy (10 times): scenario 1 and majority voting approach. *D*0: healthy state of the structure; *D*1, *D*2, and *D*3: added masses at the positions indicated in Figure 4.

	*D*0	*D*1	*D*2	*D*3
Mean	100.00	100.00	99.80	99.40
Standard deviation	0.00	0.00	0.42	0.84
Standard error	0.00	0.00	0.13	0.27

**Table 10:** Repeatability of the SHM strategy (10 times): scenario 1 and sum of the inverse distances approach. *D*0: healthy state of the structure; *D*1, *D*2, and *D*3: added masses at the positions indicated in Figure 4.

	*D*0	*D*1	*D*2	*D*3
Mean	100.00	100.00	99.80	100.00
Standard deviation	0.00	0.00	0.42	0.00
Standard error	0.00	0.00	0.13	0.00

**Table 11:** Repeatability of the SHM strategy (10 times): scenario 2 and majority voting approach. *D*0: healthy state of the structure; *D*1, *D*2, and *D*3: added masses at the positions indicated in Figure 4.

	*D*0	*D*1	*D*2	*D*3
Mean	100.00	98.10	99.70	100.00
Standard deviation	0.00	2.33	0.67	0.00
Standard error	0.00	0.74	0.21	0.00

**Table 12:** Repeatability of the SHM strategy (10 times): scenario 2 and sum of the inverse distances approach. *D*0: healthy state of the structure; *D*1, *D*2, and *D*3: added masses at the positions indicated in Figure 4.

	*D*0	*D*1	*D*2	*D*3
Mean	100.00	99.90	100.00	99.90
Standard deviation	0.00	0.32	0.00	0.32
Standard error	0.00	0.10	0.00	0.10

## Abbreviations

The following abbreviations are used in this manuscript:

CD	contrastive divergence
FFT	fast Fourier transform
ISOMAP	isometric mapping
KL	Kullback–Leibler
LDA	linear discriminant analysis
MCGS	mean-centered group scaling
NN	neural network
PCA	principal component analysis
PPV	positive predictive value
P-*t*-SNE	parametric *t*-distributed stochastic neighbor embedding
PZT	piezoelectric transducers
RBM	restricted Boltzmann machine
SHM	structural health monitoring
TNR	true negative rate
TPR	true positive rate
*t*-SNE	*t*-distributed stochastic neighbor embedding

## References

[1] Worden K., Cross E. (2018). On switching response surface models, with applications to the structural health monitoring of bridges. Mech. Syst. Sig. Process..

[2] Liu Y., Kim S.B., Chattopadhyay A., Doyle D. (2011). Application of system-identification techniques to health monitoring of on-orbit satellite boom structures. J. Spacecraft Rockets.

[3] Sohn H., Farrar C.R., Hunter N.F., Worden K. (2001). Structural health monitoring using statistical pattern recognition techniques. J. Dyn. Sys. Meas. Control.

[4] Gui G., Pan H., Lin Z., Li Y., Yuan Z. (2017). Data-driven support vector machine with optimization techniques for structural health monitoring and damage detection. KSCE J. Civ. Eng..

[5] Jimenez L.O., Landgrebe D.A. (1998). Supervised classification in high-dimensional space: geometrical, statistical, and asymptotical properties of multivariate data. IEEE Trans. Syst. Man Cybern. Part C Appl. Rev..

[6] Tibaduiza D., Mujica L., Rodellar J. (2013). Damage classification in structural health monitoring using principal component analysis and self-organizing maps. Struct. Control Health Monit..

[7] Caggiano A. (2018). Tool wear prediction in Ti-6Al-4V machining through multiple sensor monitoring and PCA features pattern recognition. Sensors.

[8] Sharma A., Paliwal K.K., Onwubolu G.C. (2006). Class-dependent PCA, MDC and LDA: A combined classifier for pattern classification. Pattern Recognit..

[9] Elvira M., Iáñez E., Quiles V., Ortiz M., Azorín J.M. (2019). Pseudo-online BMI based on EEG to detect the appearance of sudden obstacles during walking. Sensors.

[10] Jeong M., Choi J.H., Koh B.H. (2014). Isomap-based damage classification of cantilevered beam using modal frequency changes. Struct. Control Health Monit..

[11] Ullah S., Jeong M., Lee W. (2018). Nondestructive inspection of reinforced concrete utility poles with ISOMAP and random forest. Sensors.

[12] Van Der Maaten L., Hinton G.E. (2008). Visualizing data using t-SNE. J. Mach. Learn. Res..

[13] Zhang X., Gou L., Li Y., Feng J., Jiao L. (2013). Gaussian process latent variable model based on immune clonal selection for SAR target feature extraction and recognition. J. Infrared Millim. Waves.

[14] Kebede T.M., Djaneye-Boundjou O., Narayanan B.N., Ralescu A., Kapp D. Classification of malware programs using autoencoders based deep learning architecture and its application to the microsoft malware classification challenge (big 2015) dataset. Proceedings of the 2017 IEEE National Aerospace and Electronics Conference (NAECON).

[15] Li Y., Wang Y., Zi Y., Zhang M. (2015). An enhanced data visualization method for diesel engine malfunction classification using multi-sensor signals. Sensors.

[16] Balamurali M., Melkumyan A. *t*-SNE based visualisation and clustering of geological domain. Proceedings of the International Conference on Neural Information Processing.

[17] Peng Z., Cao C., Liu Q., Pan W. (2013). Human walking pattern recognition based on KPCA and SVM with ground reflex pressure signal. Math. Probl. Eng..

[18] Van Der Maaten L. Learning a parametric embedding by preserving local structure. Proceedings of the 12th International Conference on Artificial Intelligence and Statistics.

[19] Agis D., Pozo F. (2019). A frequency-based approach for the detection and classification of structural changes using t-SNE. Sensors.

[20] Westerhuis J.A., Kourti T., MacGregor J.F. (1999). Comparing alternative approaches for multivariate statistical analysis of batch process data. J. Chemom. A J. Chemom. Soc..

[21] Pozo F., Vidal Y., Salgado Ó. (2018). Wind turbine condition monitoring strategy through multiway PCA and multivariate inference. Energies.

[22] Smolensky P., Rumelhart D.E., McClelland J.L. (1986). Information processing in dynamical systems: foundations of harmony theory. Parallel Distributed Processing: Explorations in the Microstructure of Cognition, Foundations.

[23] Hinton G.E. (2012). A practical guide to training restricted Boltzmann machines. Neural Networks: Tricks of the Trade.

[24] Hinton G.E. (2002). Training products of experts by minimizing contrastive divergence. Neural Comput..

[25] Orfanidis S.J. (1995). Introduction to Signal Processing.

[26] Hossin M., Sulaiman M. (2015). A review on evaluation metrics for data classification evaluations. Int. J. Data Min. Knowl. Manage. Process.

[27] Krüger F. (2016). Activity, Context, and Plan Recognition with Computational Causal Behaviour Models. Ph.D. Thesis.

[28] Hameed N., Hameed F., Shabut A., Khan S., Cirstea S., Hossain A. (2019). An intelligent computer-aided scheme for classifying multiple skin lesions. Computers.

